# Analysis of Post-Traumatic Brain Injury Gene Expression Signature Reveals Tubulins, *Nfe2l2, Nfkb, Cd44, and S100a4* as Treatment Targets

**DOI:** 10.1038/srep31570

**Published:** 2016-08-17

**Authors:** Anssi Lipponen, Jussi Paananen, Noora Puhakka, Asla Pitkänen

**Affiliations:** 1 Epilepsy Research Laboratory, A. I. Virtanen Institute for Molecular Sciences, University of Eastern Finland, PO Box 1627, FIN-70211 Kuopio, Finland; 2 Institute of Biomedicine, University of Eastern Finland, Finland; 3 University of Eastern Finland Bioinformatics Center, University of Eastern Finland, Finland

## Abstract

We aimed to define the chronically altered gene expression signature of traumatic brain injury (TBI-sig) to discover novel treatments to reverse pathologic gene expression or reinforce the expression of recovery-related genes. Genome-wide RNA-sequencing was performed at 3 months post-TBI induced by lateral fluid-percussion injury in rats. We found 4964 regulated genes in the perilesional cortex and 1966 in the thalamus (FDR < 0.05). TBI-sig was used for a LINCS analysis which identified 11 compounds that showed a strong connectivity with the TBI-sig in neuronal cell lines. Of these, celecoxib and sirolimus were recently reported to have a disease-modifying effect in *in vivo* animal models of epilepsy. Other compounds revealed by the analysis were BRD-K91844626, BRD-A11009626, NO-ASA, BRD-K55260239, SDZ-NKT-343, STK-661558, BRD-K75971499, ionomycin, and desmethylclomipramine. Network analysis of overlapping genes revealed the effects on tubulins (*Tubb2a, Tubb3, Tubb4b), Nfe2l2, S100a4, Cd44, and Nfkb2*, all of which are linked to TBI-relevant outcomes, including epileptogenesis and tissue repair. Desmethylclomipramine modulated most of the gene targets considered favorable for TBI outcome. Our data demonstrate long-lasting transcriptomics changes after TBI. LINCS analysis predicted that these changes could be modulated by various compounds, some of which are already in clinical use but never tested in TBI.

Traumatic brain injury (TBI) affects 2.5 million people in Europe and USA, and leads to chronic disabilities in over 40% of affected patients^1^,^2^,^3^. Despite a large number of preclinical and clinical studies, there are currently no pharmacotherapies that improve post-TBI outcome^4^,^5^,^6^, and treatment of TBI remains a major unmet clinical need. The current lack of pharmacotherapies relates to our limited understanding of the molecular changes occurring at the acute, subacute, and chronic phases that could serve as stage-dependent treatment targets for this heterogeneous condition^7^.

Genome-wide gene expression studies performed to date have largely focused on analysis of the perilesional cortex and ipsilateral hippocampus^8^,^9^,^10^, and only one study has reported findings from the thalamus^11^. Almost all studies have exclusively assessed the transcriptomics changes at the acute post-TBI phase, i.e., within 24–48 h post-injury^8^,^10^,^12^,^13^,^14^,^15^,^16^,^17^,^18^,^19^,^20^,^21^,^22^,^23^,^24^,^25^,^26^,^27^,^28^,^29^, and therefore provide limited information about the mechanisms that underlie functional recovery and epileptogenesis, which occur weeks to months post-TBI^30^. Some studies demonstrated changes in the genes involved in inflammation^8^,^10^,^16^ and apoptosis^9^, most likely injury-related molecular changes. *In silico* analyses of the transcriptomics data, a strategy used to evaluate and identify new drug candidates, e.g., for the treatment of cancer^31^,^32^, have not yet been performed to reveal novel treatment targets for TBI.

To gain a better understanding of the chronic post-TBI molecular changes that could serve as treatment targets to combat post-TBI impairment, we first analyzed the transcriptomics signature of the TBI (TBI-sig) in the perilesional cortex, as well as in the ipsilateral thalamus and hippocampus in a clinically relevant animal model of closed-head TBI at 3 months after trauma. The datasets were applied to identify novel drug candidates by performing a Library of Integrated Cellular Signatures (LINCS) analysis on the TBI-sig.

## Results

### GW transcriptomics profile of the perilesional cortex, thalamus, and hippocampus after TBI

**Figure 1:**
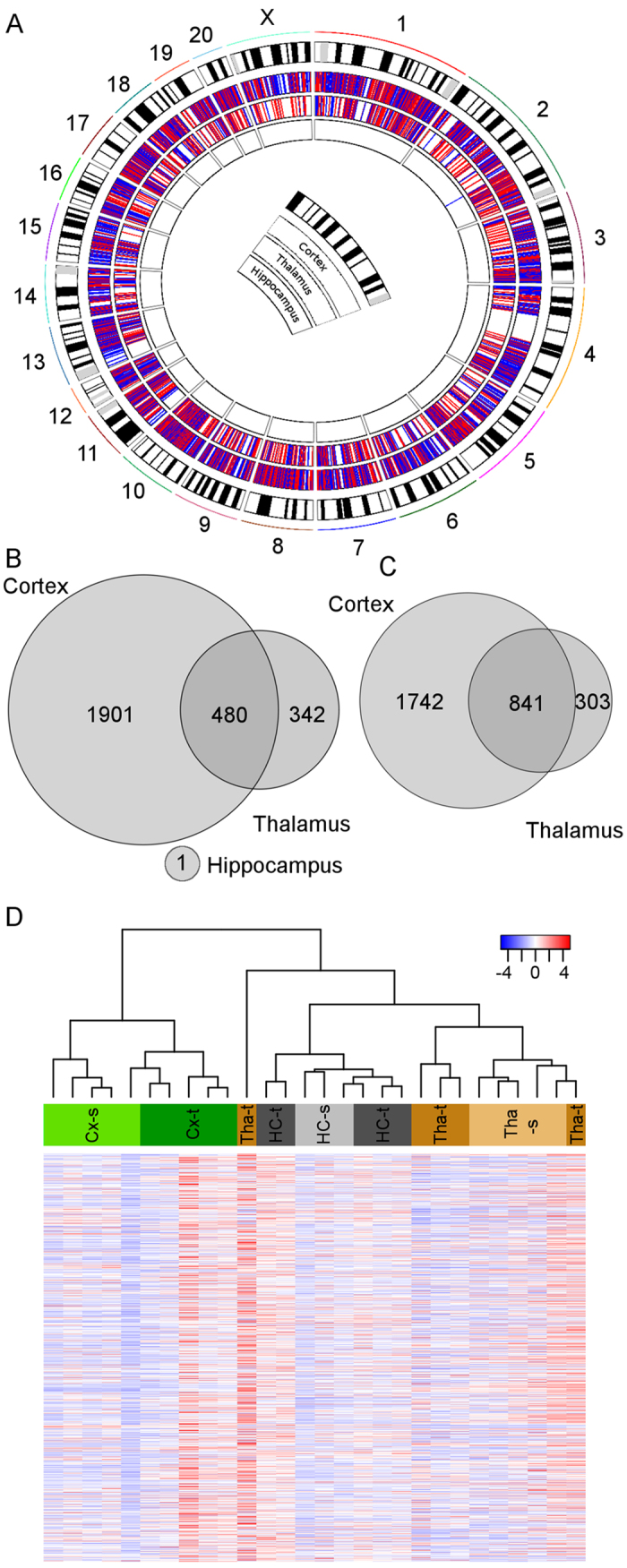
Transcriptomics profile of the perilesional cortex, ipsilateral thalamus, and ipsilateral hippocampus at 3 months after lateral fluid-percussion-induced traumatic brain injury (TBI) in rat. **(A)** A circos plot showing the chromosomal location of differentially expressed genes in the cortex (outermost circle), thalamus (middle), and hippocampus (innermost). We found 4964 differentially expressed genes in the cortex, 1966 in the thalamus, and only 1 in the hippocampus. Red lines indicate upregulated and blue lines downregulated genes. **(B)** Venn-diagram showing the number of common downregulated genes in the perilesional cortex, thalamus, and hippocampus. Altogether, 480 genes were downregulated in both the perilesional cortex and ipsilateral thalamus. **(C)** Venn-diagram showing the number of common upregulated genes. Altogether, 841 genes were upregulated in both the perilesional cortex and ipsilateral thalamus. Note the absence of any upregulated genes in the ipsilateral hippocampus. **(D)** Hierarchically clustered heatmap of differentially expressed genes in the perilesional cortex, thalamus, and hippocampus. Sham-operated and injured animals clustered into their own clusters. Also, the perilesional cortex, thalamus, and hippocampus clustered into their own clusters. Thalamic expression in one rat with TBI, however, clustered into its own branch. *Abbreviations*: Cx-s, cortex sham-operated animal; Cx-t, cortex TBI; HC-s, hippocampus sham-operated animals; HC-t, hippocampus TBI; Tha-s, thalamus sham-operated animal; Tha-t, thalamus TBI.

Three months after TBI, differential expression of 4964 genes in the perilesional cortex (2583 upregulated and 2381 downregulated) and 1966 in the thalamus (1144 upregulated and 822 downregulated) was observed compared to sham-operated animals (Fig. 1A). In the hippocampus, only one gene, *Npy2r*, was downregulated. In the perilesional cortex and thalamus, we found 480 commonly upregulated (Fig. 1B) and 841 commonly downregulated genes (Fig. 1C). The hippocampus shared no common alterations with the other brain areas.

Unsupervised hierarchal clustering of the gene expression profile in the perilesional cortex and thalamus, but not in the hippocampus, differentiated the injured rats from the control animals. Clustering also differentiated the gene expression profile of the three brain areas from each other in both controls and rats with TBI (Fig. 1D). Moreover, Spearman correlation of the fold-change in the expression of all genes between the brain areas revealed a similar direction in the gene expression between the cortex and thalamus (r_s_ = 0.4556922, p < 2.2e–16), the cortex and hippocampus (r_s_ = 0.254917, p < 2.2e–16), and the thalamus and hippocampus (r_s_ = 0.216593, p < 2.2e–16).

**Figure 2:**
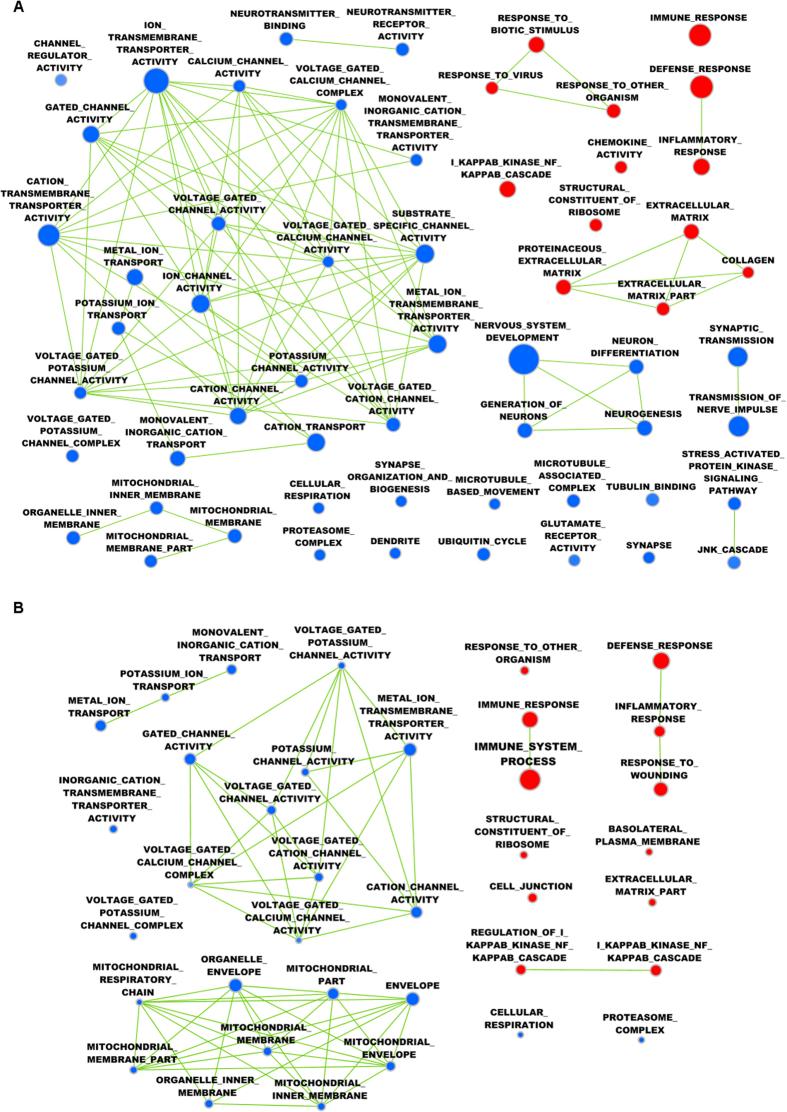
Cytoscape interaction maps of significantly enriched gene ontology terms in the **(A)** perilesional cortex and **(B)** ipsilateral thalamus at 3 months after traumatic brain injury (TBI). Interaction map from the cortex highlighted upregulation of immunity and inflammatory gene sets, and downregulation of an ion channel gene set. The same gene sets were also highlighted in the thalamus, which had enriched downregulated mitochondrion-related gene sets. Color codes: upregulated gene sets are shown in red and downregulated gene sets are shown in blue.

Gene ontology analysis indicated 57 enriched gene sets in the perilesional cortex (Fig. 2A; Supplementary Table S1), 37 in the thalamus (Fig. 2B; Supplementary Table S2), and 1 in the hippocampus. In the perilesional cortex and thalamus, ion-channel and mitochondrial gene sets were significantly downregulated, and immunity and inflammatory gene sets were upregulated. In the hippocampus, only a ligand-gated channel activity gene set was downregulated (note that the GSEA analysis was done with normal p-values to rank all genes involved, also those which were not significantly regulated after TBI).

### Generation of gene lists for network analysis

Parallel and statistically significant gene expression alterations of the perilesional cortex and thalamus were used to construct the TBI-sig. The hippocampal data were not included due to the scarcity of expression changes. Consequently, a total of 874 upregulated and 464 downregulated genes were included in the TBI-sig.

The COMPOUND-sig of nine novel top hits and two previously tested compounds in NEU cell lines were downloaded with LINCS API (http://api.lincscloud.org/). Table 1 summarizes the analysis of the overlap between the TBI-sig and COMPOUND-sig. The number of genes with significant expression changes varied from 10 to 28. All 11 overlapping gene lists were included in the IPA gene network analysis (see below).

### IPA gene network analysis of overlapping gene lists

#### Compounds that have demonstrated disease-modifying effects on epileptogenesis

Comparison of TBI-sig and COMPOUND-sig with LINCS analysis revealed two compounds, sirolimus and celecoxib, which were previously investigated in *in vivo* epileptogenesis models (see Pitkänen *et al*.^30^). The sirolimus gene networks in the IPA was generated from 20 genes (Table 1), and resulted in the generation of two networks (Supplementary Fig. S9). Many of the genes in the sirolimus network connected to the ***Ywhaz*** gene, which is upregulated by sirolimus (downregulated in the perilesional cortex and thalamus by TBI, as expected based on the negative LINCS connectivity score, Table 1). IPA gene network analysis also suggested a strong link to the ***Esr1***and ***Ubc*** genes in the sirolimus network, even though their transcription is not regulated by sirolimus or by TBI.

The celecoxib network was generated from 23 genes (Table 1), from which IPA generated 3 gene networks (Supplementary Fig. S10). Celecoxib network 1 highlighted *Nfe2l2* and *S100a4*. Celecoxib network 2 indicated a direct connection between *Tubb2a* and *Tubb3*. Both were downregulated by celecoxib in the NEU cell line, indicating a parallel effect of TBI as predicted by the positive LINCS connectivity score. Celecoxib network 3 highlighted the chromosome 16 open reading frame 45 protein-coding gene (C16of45).

#### Novel compounds revealed by LINCS analysis

**Figure 3:**
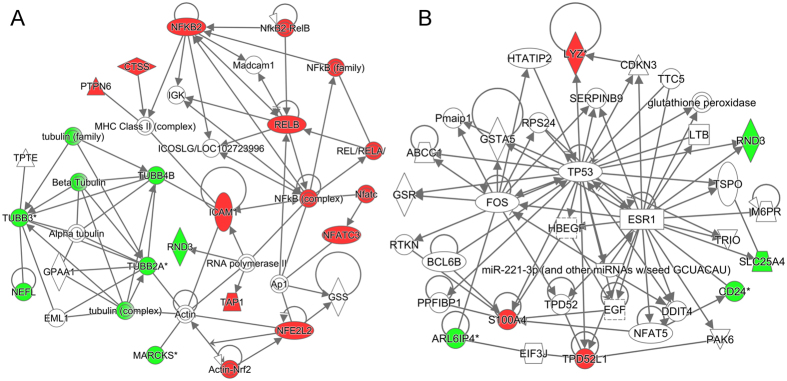
IPA gene networks generated from overlapping genes in the desmethylclomipramine signature and TBI-sig. **(A)** Network 1 highlighted tubulin downregulation, and *NFE2L2 and NFKB2* upregulation by desmethylclomipramine. **(B)** Network 2 highlighted *S100A4* upregulation by desmethylclomipramine. Color codes: red, upregulated genes; green, downregulated genes by desmethylclomipramine.

Overlapping gene lists between the nine “novel compounds” and the TBI-sig are shown in Table 1. IPA gene network analysis of gene lists related to the six “novel compounds” with a positive connectivity score in the NEU cell line (BRD-K91844626, BRD-A11009626, NO-ASA, BRD-K75971499, ionomycin, and desmethylclomipramine) indicated tubulins in five of six networks. Interestingly, *Tubb2a* and *Tubb3* were present in five networks [BRD-K91844626, BRD-A11009626, NO-ASA, ionomycin (Supplementary Fig. S1–S4), and desmethylclomipramine (Fig. 3)]. Moreover, *Tubb2a* and *Tubb3* were directly connected with each other, and both were downregulated by the five compounds. The desmethylclomipramine and NO-ASA overlapping networks contained an even larger number of tubulin-related nodes [*Tubb2a*, *Tubb3*, *Tubb4b*, tubulin (complex), β-tubulin and tubulin (family)], and all these nodes were downregulated in the NEU cell line by desmethylclomipramine and NO-ASA. Desmethylclomipramine also upregulated *Nfkb2*, *Nfe2l2*, and *S100a4.* BRD-K75971499 was the only positively connected “novel compound” without any tubulin nodes in the overlapping network (Supplementary Fig. S5). BRD-K75971499 downregulated *Vim* and upregulated *Bag3*, both of which also had a large number of connections to other genes in the overlapping network.

IPA gene network analysis of gene lists related to the nine “novel compounds” revealed that three of these compounds had a negative connectivity score in the NEU cell line (BRD-K55260239, SDZ-NKT-343, and STK-661558). All of these compounds downregulated *Cd44*, which was upregulated by TBI. The compounds exhibited no common patterns regarding gene expression upregulation (Supplementary Fig. S6-S8).

## Discussion

The present study was designed to test the hypothesis that a bioinformatics comparison of the post-TBI transcriptomic signature to a signature induced by a given drug could be used to identify novel pharmacotherapies to modify post-TBI morbidities, including epileptogenesis.

We induced TBI with lateral fluid percussion injury (FPI) in rats and performed GW mRNA-sequencing from the perilesional cortex, thalamus, and hippocampus at 3 months post-TBI. We performed the analysis at a chronic time-point to eliminate transcriptomics changes related to the acute post-injury phase and to reveal expression changes relevant to mechanisms related to chronic evolution of post-TBI morbidities, including epileptogenesis. We found major similarities in the gene expression patterns in the perilesional cortex and thalamus after TBI, but not in the hippocampus. In particular, we found negative enrichment of ion-channel and mitochondrial membrane gene sets, and positive enrichment of inflammation-related gene sets in the perilesional cortex and ipsilateral thalamus. Similar gene ontology enrichments, particularly those related to inflammation, have been reported at acute time-points after lateral FPI as well as in other experimental models of TBI^8^,^10^,^16^,^33^,^34^; thus, our data indicate that these functional gene sets remain activated over a wide post-TBI time window. Our data also revealed a strong downregulation of ion channel-related gene sets. Only two previous transcriptomics studies have reported regulation of ion channels at an acute time-point (<24 h) after experimental TBI^16^,^34^, showing changes in potassium and sodium channels. Our data show a strong downregulation in a large number of ion-channel gene sets, providing a molecular basis for the long-lasting changes in excitability observed after TBI. Taken together, perilesional cortex and thalamus, which are anatomically heavily interconnected, maintain similar transcriptomics changes in inflammatory and ion channel pathways. Changes in the expression levels are chronic, lasting up to 3 months, which suggests a wide therapeutic time window for treatments targeting these gene networks.

Next we performed a LINCS analysis to determine compounds that modulate the TBI-sig. Comparison of the TBI-sig with the COMPOUND-sig of compounds listed in the LINCS database pinpointed novel TBI-sig modifying compounds, but also celecoxib and sirolimus, two treatments previously shown to have disease-modifying effects in proof-of-concept studies using *in vivo* models^35^, including post-TBI epileptogenesis^36^. In particular, we found celecoxib to “strengthen” the expression of 23 genes regulated by TBI, whereas sirolimus had an opposing (“normalizing”) effect on the expression of 20 genes regulated by TBI.

Celecoxib upregulated transcription factor *Nfe2l2* (or *Nrf2*), which is translocated to the nucleus after brain injuries^37^,^38^ to promote the expression of numerous antioxidant, anti-inflammatory, and neuroprotective proteins^37^,^39^,^40^. Moreover, a recent study reported that mice injected with AAV Nrf2 displayed significantly fewer generalized seizures, with a profound reduction in microglia activation^41^. Celecoxib also upregulated *S100a4*, which is normally expressed in the brain at low levels^42^. *S100a4* induces neuroprotection in models of brain injury^43^. Moreover, our analysis revealed that celecoxib downregulated *Tubb3* and *Tubb2a*. This is of interest as inactivating mutations in genes encoding tubulins were recently reported to associate with epilepsy, and abnormal neuronal migration and organization^44^,^45^. While the effects of celecoxib on *Nfe2l2* and *S100a4* support the endogenous chronic repair mechanisms of the brain, it remains to be explored whether or not the net effect of tubulin downregulation on post-TBI recovery is favorable.

The LINCS analysis also highlighted an mTOR inhibitor, sirolimus (rapamycin), as a drug with a high negative connectivity score. Sirolimus is the most investigated compound in different genetic and acquired models of epileptogenesis, showing favorable antiepileptogenic and co-morbidity modifying effects (for review, see Pitkänen *et al*.^30^). Its gene expression profile, however, opposed the changes induced by TBI. Also, the gene networks regulated by sirolimus showed little overlap with those regulated by celecoxib, supporting the concept that post-injury outcome can be modified by affecting complementary non-overlapping molecular networks.

Next we investigated whether the compounds affecting the TBI-sig had any common gene targets, and whether the targets are the same as those modulated by known antiepileptogenic compounds. From the 1064 novel drug candidates found by the LINCS analysis, desmethylchlomipramine and NO-ASA showed the most remarkable modulatory effects on the post-TBI transcriptomic changes. Similar to celecoxib, desmethylclomipramine upregulated *Nfe2l2*, *Nfkb2*, and *S100a4*, and downregulated *tubulins*. NO-ASA (3-nitrooxyphenyl acetylsalicylate, NCX-4016), an anti-inflammatory and antithrombotic compound^46^, modulated the expression of 28 genes in the TBI-sig, including downregulation of tubulin expression. Ionomycin, an ionophore known to increase intracellular Ca^2+^ 47, modulated both *Nfe2l2* and tubulins, whereas BRD-K91844626 and BRD-A11009626 modulated only tubulins. The four remaining compounds with unknown mechanisms of action, BRD-K55260239, SDZ-NKT343, STK-661558, and BRD-K75971499 all downregulated *Cd44*, which encodes a cell-surface glycoprotein involved in cell adhesion and migration^48^, and remains upregulated for up to 2 months in a mouse model of stab brain injury^49^. These compounds exhibited no overlap with the gene networks modulated by celecoxib, sirolimus, desmethylchlomipramine, or ionomycin.

Taken together, our *in silico* data highlighted tubulins, *Nfe2l2*, *Nfkb2*, and *S100a4* as target genes modulated by compounds with a high LINCS connectivity score relative to the TBI-sig. Moreover, our data suggested that desmethylclomipramine, an active metabolite of the commonly used antidepressant clomipramine, is a promising candidate to be explored as a recovery-enhancing treatment after brain injury.

## Materials and Methods

### Animals

TBI was induced in five adult male Sprague-Dawley rats (330–370 g at the time of TBI or sham operation) with lateral FPI having an impact pressure 3.30 ± 0.01 atm (duration of post-impact apnea 15–25 sec) as previously described^50^,^51^. Five sham-operated rats served as controls. At 3 months after TBI, rats were deeply anesthetized with isoflurane and decapitated. The brain was removed from the skull, flushed with 0.9% cold (4 °C) sodium chloride, and two 2-mm-thick coronal slices (between −2.2 to −6.2 from the bregma) were cut with a slicing matrix (#15007, Rodent Brain Matrix, Ted Pella, Inc, Redding, CA, USA). The perilesional cortex, thalamus, and dentate gyrus (including CA3c-b) were then dissected under a magnifying glass on top of the light table, moved to a microcentrifuge tube, snap frozen in liquid nitrogen, and stored at −70 °C until RNA extraction. The remaining tissue pieces were immersion-fixed in 10% formalin and thionin-stained to verify the occurrence of lesion (data not shown).

All animal procedures were approved by The Animal Ethics Committee of Provincial Government of Southern Finland and performed in accordance with the guidelines of the European Community Council Directives 2010/63/EU.

### Preparation of the sequencing library and RNA-sequencing

RNA was extracted from the perilesional cortex, thalamus, and hippocampus DNaesy Blood & Tissue kit (#69504, Qiagen, Hilden, Germany) followed by DNase digestion. mRNA from 2 μg of total RNA was enriched using Dynabeads Oligo (dT)_25_ beads (#61002, Invitrogen, Carlsbad, CA, USA). The sequencing library was prepared with the NEBNext mRNA Library Prep Reagent Set (#E6100S, New England Biolabs, Ipswich, MA, USA). Quality control of the total RNA and sequencing libraries was performed using a MultiNA electrophoresis device (Shimazu, Kyoto, Japan). Then, sequencing of the mRNA library (RNA-Seq) for the perilesional cortex and hippocampus was carried out with an Illumina Genome Analyzer IIx (San Diego, CA, USA) with 36 cycles, and for the thalamus using Illumina HiSeq 2000 (San Diego, CA, USA) with 50 cycles. Base-calling was performed using an Illumina Off-Line Basecaller v1.8. RNA-Seq raw data were saved to the NCBI Gene Expression Omnibus (GEO; series accession number GSE80174).

### Bioinformatics

Quality control of the sequencing raw reads was performed using FastQC^52^. Sequencing reads were aligned to the Ensemble RN5 genome with Spliced Transcripts Alignment to Reference (STAR) software (version 2.3.0e_r291)^53^. Two hippocampal samples were discarded from further analysis due to low alignment percentage (32.9% and 35.5%). From the remaining 28 samples, 64.3 ± 7.9% of the perilesional cortical (5 control, 5 TBI), 66.9 ± 3.7% hippocampal (3 control, 5 TBI), and 80.5 ± 4.6% of thalamic (5 control, 5 TBI) raw reads were aligned to the RN5 reference genome. Differentially expressed genes were identified with DEseq2 R package (R version 3.1.0)^54^. The adjusted p-value was calculated with a Benjamini–Hochberg false discovery rate (FDR). FDR < 0.05 was considered a statistically significant difference in the gene expression. Hierarchal clustering was performed by heatmap.2 function in gplots R package to genes that had FDR < 0.05 in at least one brain area. Direction of the fold-change between the brain areas was compared with Spearman’s correlation by corr.test R function.

Enrichment of gene ontology terms was analyzed with Gene Set Enrichment Analysis (GSEA)^55^ with C5-catogory gene sets in Molecular Signatures Database (MSigDB). Ranked-gene lists for GSEA of the perilesional cortex, hippocampus, and thalamus were prepared by ranking the genes in order by p-value and then multiplying with the sign of the fold-change. Upregulated genes were assigned with positive and downregulated genes with a negative rank. The GSEA gene set was considered significantly enriched when the q-value was <0.01. GSEA results were visualized with a enrichment map v.1.3 plugin with P-value Cutoff 0.005; q-value Cutoff 0.01 and Similarity Cutoff 0.5 in Cytoscape 2.8.3^56^.

### Evaluation of drug candidates

We first generated the “TBI gene expression signature” (TBI-sig) from our TBI dataset, which included genes with significantly altered expression in both the cortex and thalamus, and submitted it to the LINCS web query. This TBI-sig was compared to the expression signature of each test compound (COMPOUND-sig) separately in three cell lines: in terminally differentiated neurons (NEU), terminally differentiated neurons treated with KCl (NEU.KCL), and iPS-derived neural progenitor cells (NPC), which were available in the LINCS database. To filter reproducible gene expression signatures from all query results and fetch differentially expressing genes by compounds, we used the Lincscloud Application Programming Interface (API) through a custom R-script. This resulted in a total of 3358 hits with a connectivity score between 1.0 to −1.0. The positive connectivity score in the LINCS analysis indicated that the direction (either upregulated or downregulated) of the TBI-sig was similar to that of the COMPOUND-sig; a negative connectivity score indicated a reverse direction, and a score of 0 indicated no association between the expression signatures. Drug candidates with a connectivity score from 0.1 to −0.1 were filtered out from the final list as their connectivity to the TBI-sig was considered weak. This resulted in data from 1064 compounds, of which sirolimus and celecoxib demonstrate favorable disease-modifying effects on epileptogenesis in *in vivo* animal models. Carbamazapine, with no documented effect on epileptogenesis, was also on the list^35^. We also found tacrolimus and zonisamide, included in *in vivo* epileptogenesis studies, on the original list of 3358 compounds, but their connectivity scores did not survive the filtering criterion.

Next, we prepared a list of 18 “novel top hits”, which included 3 compounds with the highest positive and 3 compounds with the highest negative connectivity scores from each of the 3 cell lines (Supplementary Table S3). Another list included the three compounds that have already been assessed in *in vivo* epileptogenesis models, i.e., sirolimus (positive effect), celecoxib (positive effect), and carbamazepine (no effect). Next, we generated the list of overlapping genes between the TBI-sig and the COMPOUND-sig induced in NEU cell lines only by the a) novel top hits (n = 9) and b) previously tested compounds (n = 2) (Table 1). As summarized in Table 1, the overlapping gene lists were available from 9 of 18 “novel compounds” and from 2 already known disease-modifying compounds, which were then used to generate gene networks with IPA.

## Additional Information

**How to cite this article**: Lipponen, A. *et al*. Analysis of Post-Traumatic Brain Injury Gene Expression Signature Reveals Tubulins, *Nfe2l2, Nfkb, Cd44, and S100a4* as Treatment Targets. *Sci. Rep.* **6**, 31570; doi: 10.1038/srep31570 (2016).

## Supplementary Material

Supplementary Information

## Figures and Tables

**Table 1:** Overlapping genes between the transcriptomics signature of the compound (COMPOUND-sig) and traumatic brain injury (TBI-sig).

Compound	Connectivity score	Number of overlapping genes	Number of IPA gene networks	Genes upregulated by the compound	Genes downregulated by the compound
BRD-K91844626	0,3421	21	2	CTSS, EHD2, GLIPR1, MMP24, NUCB2, PSMB8, TSFM, TSPAN6, TXNIP	ATP1B1, CD24, EEF1A2, MARCKS, NEFL, SHC1, SULT4A1, TPI1, TRIB2, TUBB2A, TUBB3, VIM
BRD-A11009626	0,3354	18	2	PSMB8, SLC8B1, OLR1, NFKB2, CTSS, TXNIP, GLIPR1, HLA-E,DUSP14, VAV1	CD24, CSRP2, RND3, YBX3, NEFL, TUBB3, TUBB2A, INA
NO-ASA	0,3267	23	3	SLC8B1, CACNA1G, IRAK4, LGALS9, HMOX1, TCIRG1, BNIP3, LAMP1, KCNK1, MVP, BAG3, CD37, CTSS, PLXDC1, ADAP2	CD24, CSRP2, RND3, YBX3, NEFL, TUBB3, TUBB2A, INA
BRD-K55260239	−0,3115	10	1	APPL2, SLC27A3, MUC1	LITAF,CD44, SOX10, SHC1, RGS3, SP110, ALAS1
SDZ-NKT-343	−0,3089	17	2	C16orf45, SCG5, ATP8A2, STXBP1, CIAPIN1, BAG3, BST2	IGFBP7, CD44, RIN3, EFCAB14, ALAS1 S100A6, SLC27A3, CD9, ZFP36L1, A2M,
STK-661558	−0,3079	21	2	TSPAN6, PITPNA, APPL2, ABHD4, TNFAIP2, NELL1, NECAB3, SEMA3B, COX5A	FLT3, IGFBP7, YBX3, CD44, GABBR2, MOCS1, S100A1, ITGAL, VAMP1, PGAM4, A2M, PGAM4
BRD-K75971499	0,2184	19	1	FLT3, SZRD1, HADH, HMOX1, BNIP3, GADD45B, BAG3, TPD52L1, HSPB1, ALDOA, GLIPR1	MARCKS, DDR1, ABCB9, TRIB2, RND3, CD44, CLSTN1, VIM
Ionomycin	0,2624	20	2	NFE2L2, ETV5, FH, LYZ, FAH, RASSF4, GADD45B, ICAM1, PLCB3, CTSS, FCGR2C, FCGR2B	CD24, MARCKS, FABP7, RPS4X, PABPC1, NEFL, TUBB3, TUBB2A
Desmethylclomipramine	0,2765	20	2	TAP1, NFE2L2, LYZ, S100A4, PTPN6, NFATC3, NFKB2, ICAM1, CTSS, TPD52L1, RELB	CD24, MARCKS, ARL6IP4, RND3, TUBB4B, SLC25A4, NEFL, TUBB3, TUBB2A
Sirolimus	−0,2127	17	2	LAMP2, PSMB8, CAMK2B, YWHAZ, ADCY1, HMOX1, TRAPPC6A, FAS, KCNK1, CTSS	CD24, FH, CTNNA1, MCM3, SPOCK1, GEM, A2M
Celecoxib	0,2094	21	3	TAP1, NFE2L2, LYZ, S100A4, PVRL2, KCNK1, CTSS, TPD52L1, HLA-F, GLIPR1,	CD24, FABP7, C16orf45, RPL7, EIF4EBP1, SPOCK1, NEFL,TUBB3, TUBB2A, STK25, ETNK2

Abbreviations: IPA,Ingenuity Pathaway analysis; NO-ASA, 3-Nitrooxyphenyl acetylsalicylate.
