# Supplementary material for: Analysis of Post-Traumatic Brain Injury Gene Expression Signature Reveals Tubulins, Nfe2l2, Nfkb, Cd44, and S100a4 as Treatment Targets
Source: Sci Rep. 2016 Aug 17;6:31570. doi: 10.1038/srep31570 (PMC4987651; doi:10.1038/srep31570)
Supplement: Supplementary Information [file srep31570-s1.pdf]

Analysis of Post-Traumatic Brain Injury Gene Expression Signature Reveals Tubulins, NFE2L2, NFkB, CD44, and S100A4 as Treatment Targets

Anssi Lipponen, Jussi Paananen, Noora Puhakka, Asla Pitkänen

Supplementary Table 1

Supplementary Table 1. Enriched gene ontology terms in the perilesional cortex.

Name	FDR q-val	Pos/Neg enrihment
STRUCTURAL_CONSTITUENT_OF_RIBOSOME	0	Pos
PROTEINACEOUS_EXTRACELLULAR_MATRIX	0	Pos
DEFENSE_RESPONSE	0	Pos
COLLAGEN	0	Pos
RESPONSE_TO_OTHER_ORGANISM	0	Pos
EXTRACELLULAR_MATRIX	0	Pos
EXTRACELLULAR_MATRIX_PART	0,001416899	Pos
INFLAMMATORY_RESPONSE	0,001239786	Pos
RESPONSE_TO_VIRUS	0,001229948	Pos
IMMUNE_RESPONSE	0,003123401	Pos
CHEMOKINE_ACTIVITY	0,00691114	Pos
I_KAPPAB_KINASE_NF_KAPPAB_CASCADE	0,009965185	Pos
VOLTAGE_GATED_CHANNEL_ACTIVITY	0	Neg
VOLTAGE_GATED_CATION_CHANNEL_ACTIVITY	0	Neg
GATED_CHANNEL_ACTIVITY	0	Neg
METAL_ION_TRANSMEMBRANE_TRANSPORTER_ACTIVITY	0	Neg
POTASSIUM_ION_TRANSPORT	0	Neg
CATION_CHANNEL_ACTIVITY	0	Neg
VOLTAGE_GATED_POTASSIUM_CHANNEL_ACTIVITY	1,35E-04	Neg
ION_CHANNEL_ACTIVITY	1,18E-04	Neg
VOLTAGE_GATED_CALCIUM_CHANNEL_COMPLEX	1,05E-04	Neg
SYNAPTIC_TRANSMISSION	9,44E-05	Neg
TRANSMISSION_OF_NERVE_IMPULSE	8,58E-05	Neg
METAL_ION_TRANSPORT	7,86E-05	Neg
MONOVALENT_INORGANIC_CATION_TRANSPORT	1,41E-04	Neg
POTASSIUM_CHANNEL_ACTIVITY	1,31E-04	Neg
VOLTAGE_GATED_POTASSIUM_CHANNEL_COMPLEX	1,22E-04	Neg
SUBSTRATE_SPECIFIC_CHANNEL_ACTIVITY	1,14E-04	Neg
PROTEASOME_COMPLEX	2,15E-04	Neg
SYNAPSE_ORGANIZATION_AND_BIOGENESIS	2,03E-04	Neg
VOLTAGE_GATED_CALCIUM_CHANNEL_ACTIVITY	1,92E-04	Neg
CATION_TRANSMEMBRANE_TRANSPORTER_ACTIVITY	2,73E-04	Neg
CELLULAR_RESPIRATION	8,92E-04	Neg
SYNAPSE	8,92E-04	Neg
NERVOUS_SYSTEM_DEVELOPMENT	9,66E-04	Neg
CALCIUM_CHANNEL_ACTIVITY	0,001185576	Neg
NEUROGENESIS	0,001174722	Neg
ION_TRANSMEMBRANE_TRANSPORTER_ACTIVITY	0,001199935	Neg
MICROTUBULE_ASSOCIATED_COMPLEX	0,001155493	Neg
STRESS_ACTIVATED_PROTEIN_KINASE_SIGNALING_PATHWAY	0,001369768	Neg
DENDRITE	0,001385402	Neg
NEUROTRANSMITTER_RECEPTOR_ACTIVITY	0,00157421	Neg
GENERATION_OF_NEURONS	0,001695259	Neg
GLUTAMATE_RECEPTOR_ACTIVITY	0,001867902	Neg
CATION_TRANSPORT	0,002028934	Neg
MITOCHONDRIAL_INNER_MEMBRANE	0,00196926	Neg
MICROTUBULE_BASED_MOVEMENT	0,002040412	Neg
MONOVALENT_INORGANIC_CATION_TRANSMEMBRANE_TRANSPORTER_ACTIVITY	0,002059049	Neg
MITOCHONDRIAL_MEMBRANE	0,002027047	Neg
NEURON_DIFFERENTIATION	0,002399285	Neg
NEUROTRANSMITTER_BINDING	0,002405064	Neg
MITOCHONDRIAL_MEMBRANE_PART	0,002523794	Neg
JNK_CASCADE	0,005896469	Neg
ORGANELLE_INNER_MEMBRANE	0,007973801	Neg
TUBULIN_BINDING	0,008412651	Neg
UBIQUITIN_CYCLE	0,008242112	Neg
CHANNEL_REGULATOR_ACTIVITY	0,008293908	Neg

Abbreviations: Name, Enriched gene ontology term; FDR q-val, False discovery rate q-value; Pos, positively enriched ontology term; Neg, negatively enriched ontology term.

Supplementary Table 2

Supplementary Table 2. Enriched gene ontology terms in the thalamus

NAME	FDR q-val	Pos/Neg enrihment
IMMUNE_RESPONSE	0	Pos
BASOLATERAL_PLASMA_MEMBRANE	5,62E-04	Pos
IMMUNE_SYSTEM_PROCESS	0,001459492	Pos
DEFENSE_RESPONSE	0,001373219	Pos
STRUCTURAL_CONSTITUENT_OF_RIBOSOME	0,002185657	Pos
I_KAPPAB_KINASE_NF_KAPPAB_CASCADE	0,005277846	Pos
CELL_JUNCTION	0,004680899	Pos
RESPONSE_TO_OTHER_ORGANISM	0,005468081	Pos
INFLAMMATORY_RESPONSE	0,005588763	Pos
RESPONSE_TO_WOUNDING	0,005253211	Pos
REGULATION_OF_I_KAPPAB_KINASE_NF_KAPPAB_CASCADE	0,005674641	Pos
EXTRACELLULAR_MATRIX_PART	0,007197761	Pos
POTASSIUM_ION_TRANSPORT	0	Neg
MITOCHONDRIAL_MEMBRANE	4,43E-04	Neg
MITOCHONDRIAL_MEMBRANE_PART	2,95E-04	Neg
VOLTAGE_GATED_CATION_CHANNEL_ACTIVITY	4,39E-04	Neg
VOLTAGE_GATED_CHANNEL_ACTIVITY	3,51E-04	Neg
VOLTAGE_GATED_POTASSIUM_CHANNEL_ACTIVITY	0,001032828	Neg
MITOCHONDRIAL_INNER_MEMBRANE	8,85E-04	Neg
POTASSIUM_CHANNEL_ACTIVITY	0,00122112	Neg
METAL_ION_TRANSMEMBRANE_TRANSPORTER_ACTIVITY	0,001975178	Neg
VOLTAGE_GATED_POTASSIUM_CHANNEL_COMPLEX	0,00196713	Neg
ORGANELLE_INNER_MEMBRANE	0,002372856	Neg
CELLULAR_RESPIRATION	0,002391202	Neg
MITOCHONDRIAL_ENVELOPE	0,002338413	Neg
MITOCHONDRIAL_RESPIRATORY_CHAIN	0,002234509	Neg
VOLTAGE_GATED_CALCIUM_CHANNEL_ACTIVITY	0,002626745	Neg
MITOCHONDRIAL_PART	0,004482372	Neg
PROTEASOME_COMPLEX	0,00437347	Neg
INORGANIC_CATION_TRANSMEMBRANE_TRANSPORTER_ACTIVITY	0,004326779	Neg
METAL_ION_TRANSPORT	0,006309745	Neg
GATED_CHANNEL_ACTIVITY	0,006543345	Neg
CATION_CHANNEL_ACTIVITY	0,0067354	Neg
VOLTAGE_GATED_CALCIUM_CHANNEL_COMPLEX	0,006429246	Neg
ORGANELLE_ENVELOPE	0,007948274	Neg
ENVELOPE	0,008040466	Neg
MONOVALENT_INORGANIC_CATION_TRANSPORT	0,009620455	Neg

Abbreviations: Name, Enriched gene ontology term; FDR q-val, False discovery rate q-value; Pos, positively enriched ontology term; Neg, negatively enriched ontology term.

Supplementary Table 3

Supplementary Table 3. Three highest and lowest connectivity scores in the LINCS analysis. Scores are shown separately for neuron cell lines, neuron cell lines treated with KCl, and neural progenitors cell lines.

Compound	NEU highest connectivity score	NEU lowest connectivity score	NEU.KCL highest connectivity score	NEU.KCL lowest connectivity score	NPC highest connectivity score	NPC lowest connectivity score
BRD-K91844626	0,3421	0,2078	0,2888	0,2366	nd	nd
BRD-A11009626	0,3354	0,3354	nd	nd	nd	nd
NO-ASA	0,3267	0,3267	0,3455	0,2084	0,2753	0,2463
BRD-K55260239	-0,3115	-0,3115	nd	nd	nd	nd
SDZ-NKT-343	-0,3089	-0,3089	nd	nd	nd	nd
STK-661558	-0,3079	-0,3079	nd	nd	nd	nd
BRD-K75971499	0,2184	0,2184	0,4029	0,4029	nd	nd
Ionomycin	0,2624	0,2624	0,3784	0,3784	nd	nd
BG-1024	nd	nd	0,3743	0,3743	0,2797	0,2797
Pioglitazone	nd	nd	-0,3305	-0,3305	nd	nd
Desmethylclomipramine	0,2765	0,2765	0,2867	-0,307	nd	nd
BRD-K72015216	nd	nd	-0,3012	-0,3012	nd	nd
BRD-K78075916	nd	nd	0,1948	0,1948	0,3355	0,3355
Parthenolide	nd	nd	nd	nd	0,334	0,334
XE-991	nd	nd	nd	nd	0,3263	0,3263
BRD-K46047563	nd	nd	nd	nd	-0,3097	-0,3097
Testosterone	nd	nd	nd	nd	0,2464	-0,3059
UCL-2077	nd	nd	nd	nd	-0,3004	-0,3004
Sirolimus	-0,2127	-0,2127	0,2853	0,2853	0,2888	0,2143
Celecoxib	0,2094	0,2094	0,2822	0,2822	nd	nd
Carbamazepine	nd	nd	0,3075	-0,1693	nd	nd

Abbreviations: NEU, Neuron cell lines; NEU.KCL, Neuron cell lines treated with KCl; NPC, Neural progenitors cell lines; nd, No data in LINCS database; LINCS, Library of Integrated Cellular Signatures The 3 cell lines were chosen as they (a) represent neuronal cell lines, (b) mimic increased excitability (NEU.KCL) which has previously been reported to be recovery-enhancing in TBI and other acquired brain-injury model[1–3], or (c) model developing cells, recapitulating the developmental processes that become activated after brain injury[4].

BRD-K91844626 1/2

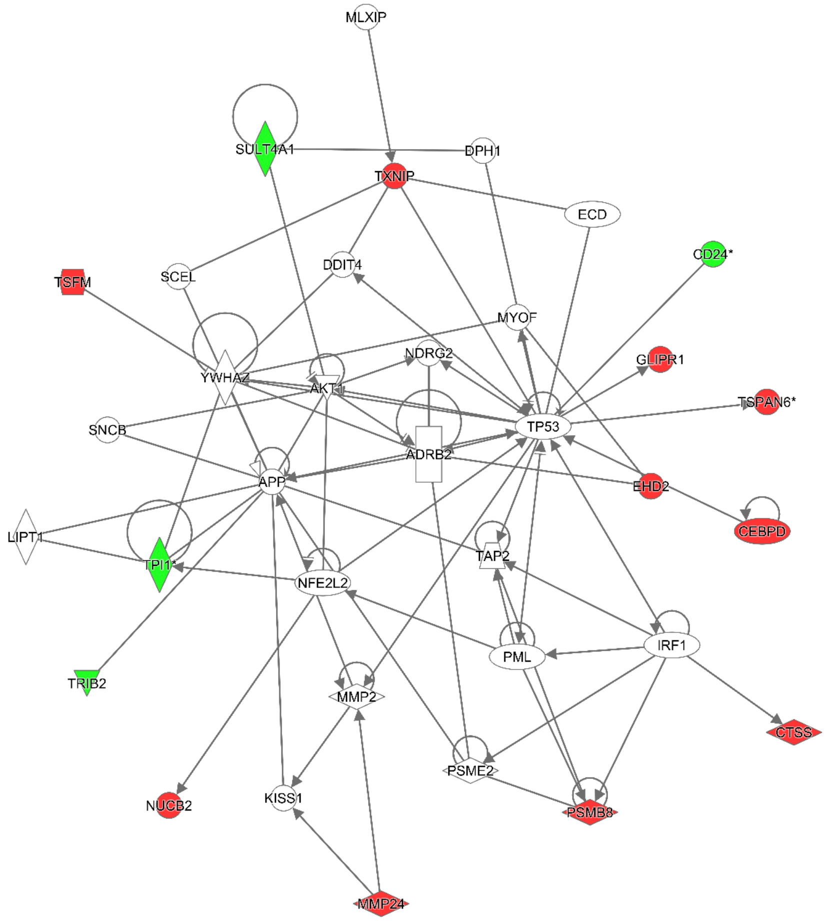

BRD-K91844626 2/2

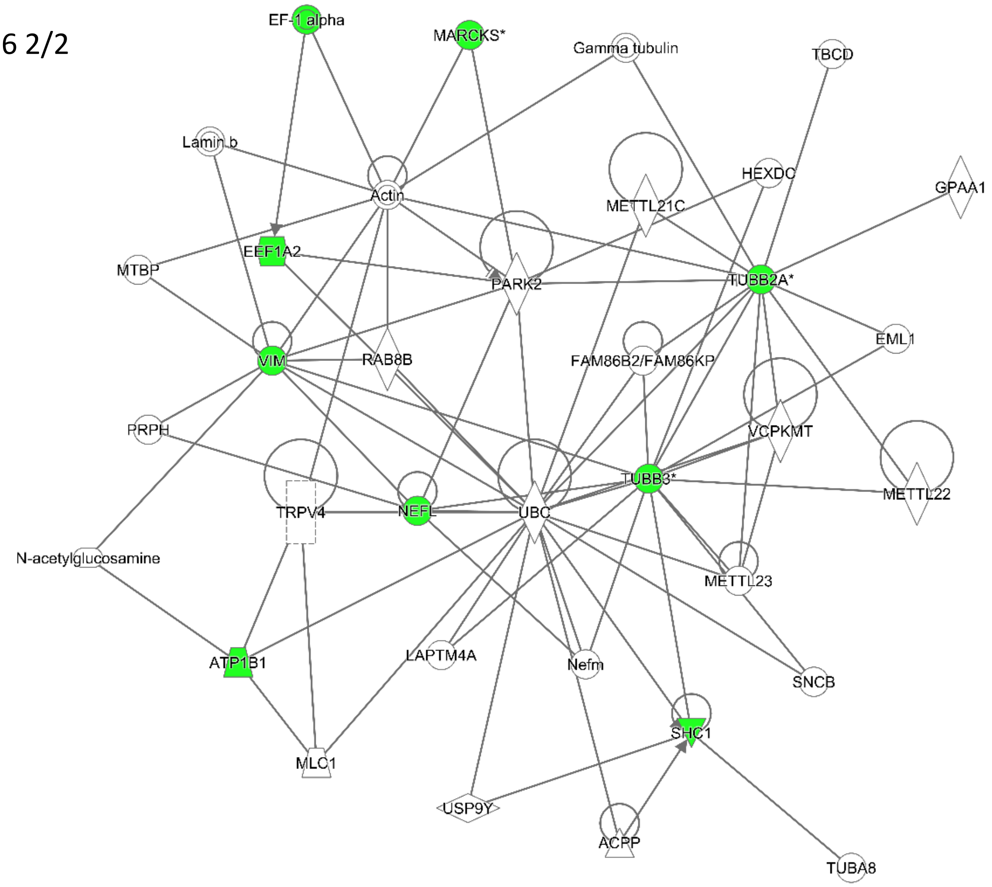

Supplementary Figure 1. IPA generated two gene networks (1/2 and 2/2) from genes overlapping in the BRD-K91844626 and TBI signatures. Network 2 highlighted downregulation of tubulins. Color codes: red, upregulated genes; green, downregulated genes by BRD-K91844626.

BRD-A11009626 1/2

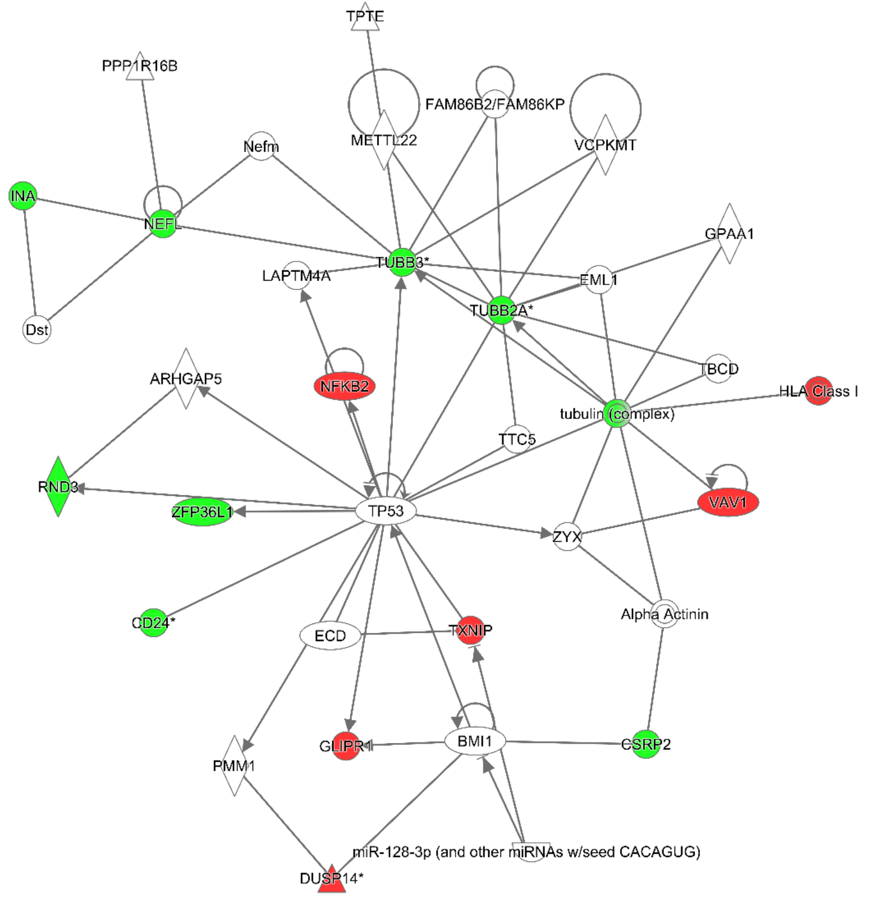

BRD-A11009626 2/2

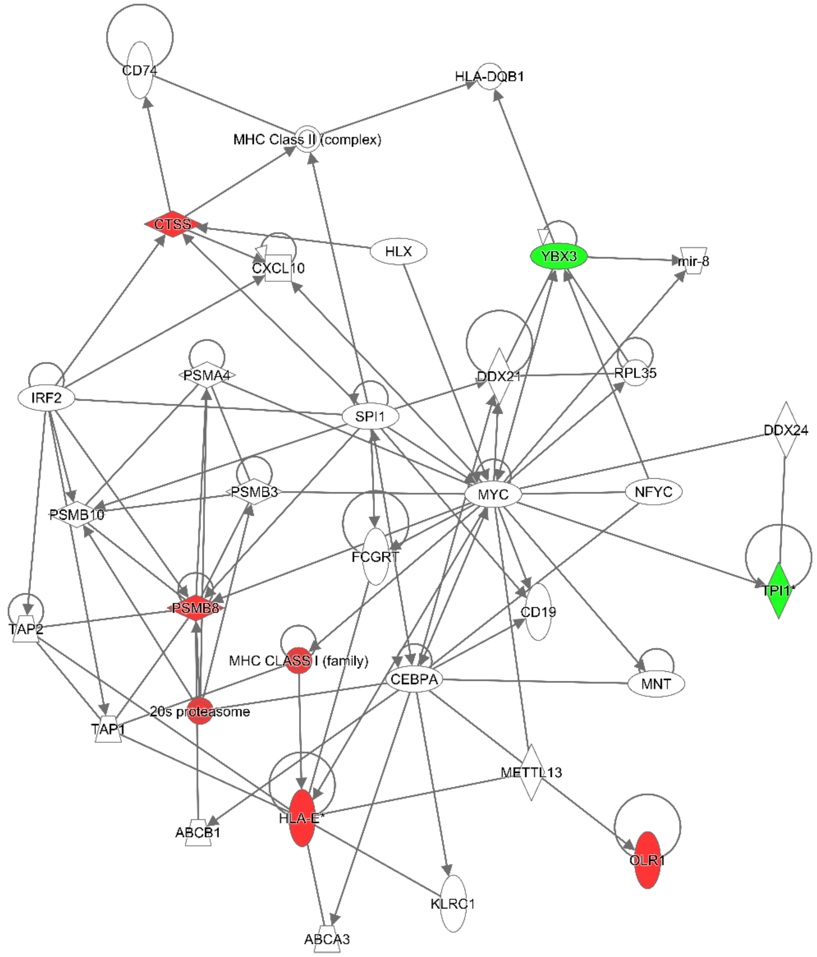

Supplementary Figure 2. IPA generated two gene networks (1/2 and 2/2) from genes overlapping in the BRD-A11009626 and TBI signatures. Network 1 highlighted downregulation of tubulins. Color codes: red, upregulated genes; green, downregulated genes by BRD-A11009626.

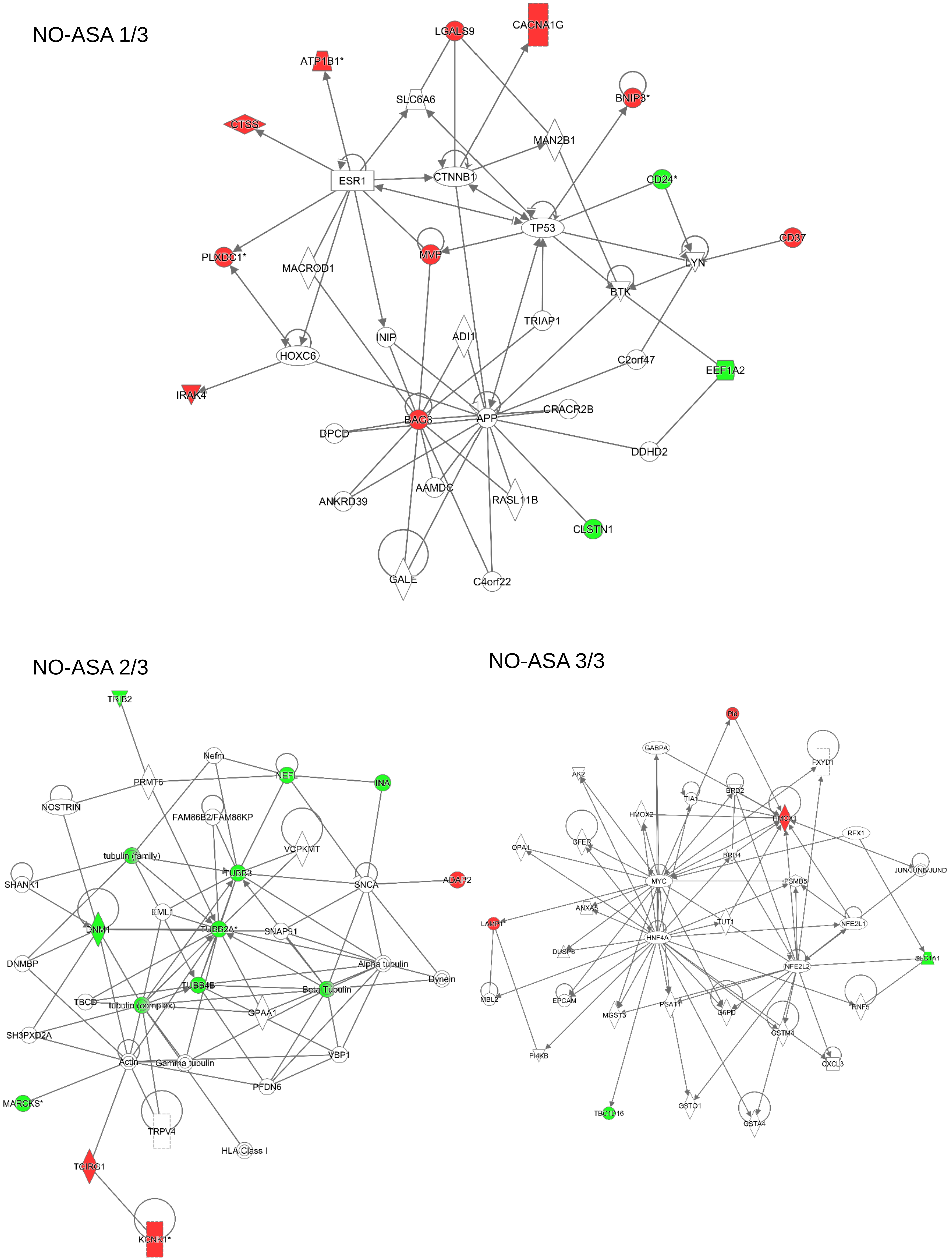

Supplementary Figure 3. IPA generated three gene networks (1/3, 2/3, and 3/3) from genes overlapping in the NO-ASA and TBI signatures. Network 2 highlighted downregulation of tubulins. Color codes: red, upregulated genes; green, downregulated genes by NO-ASA.

ionomycin 1/2

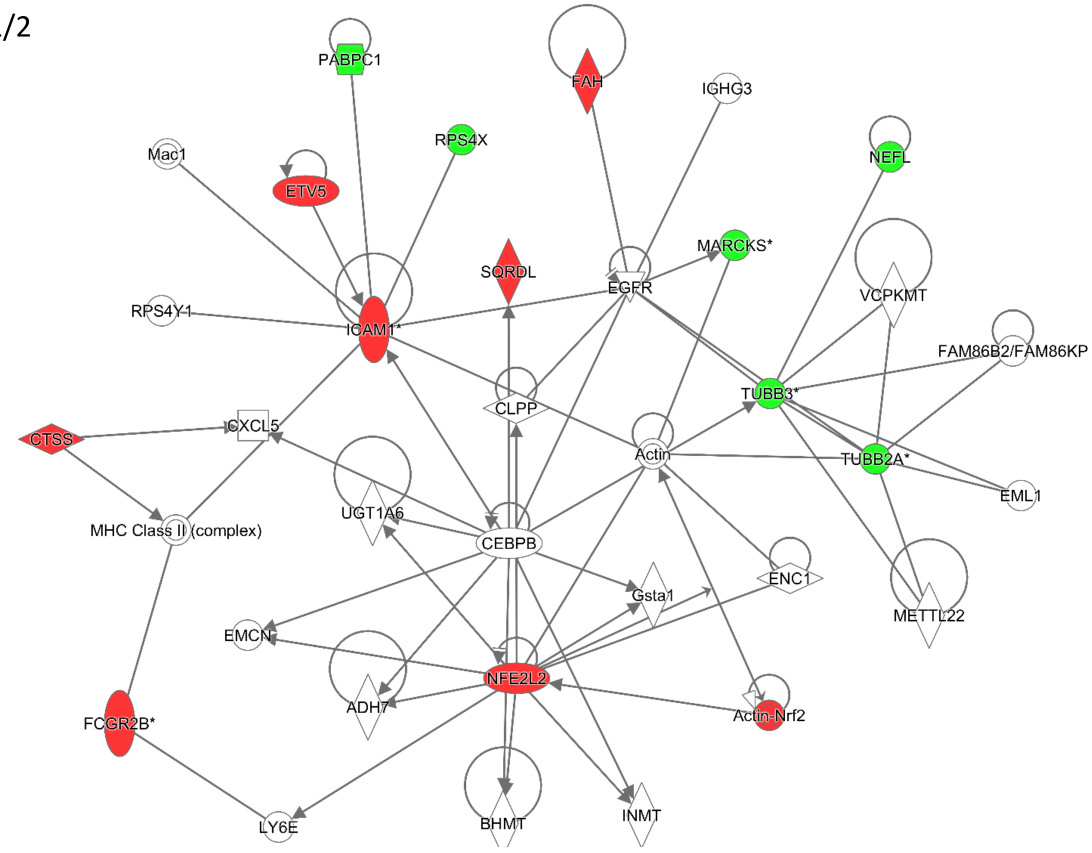

ionomycin 2/2

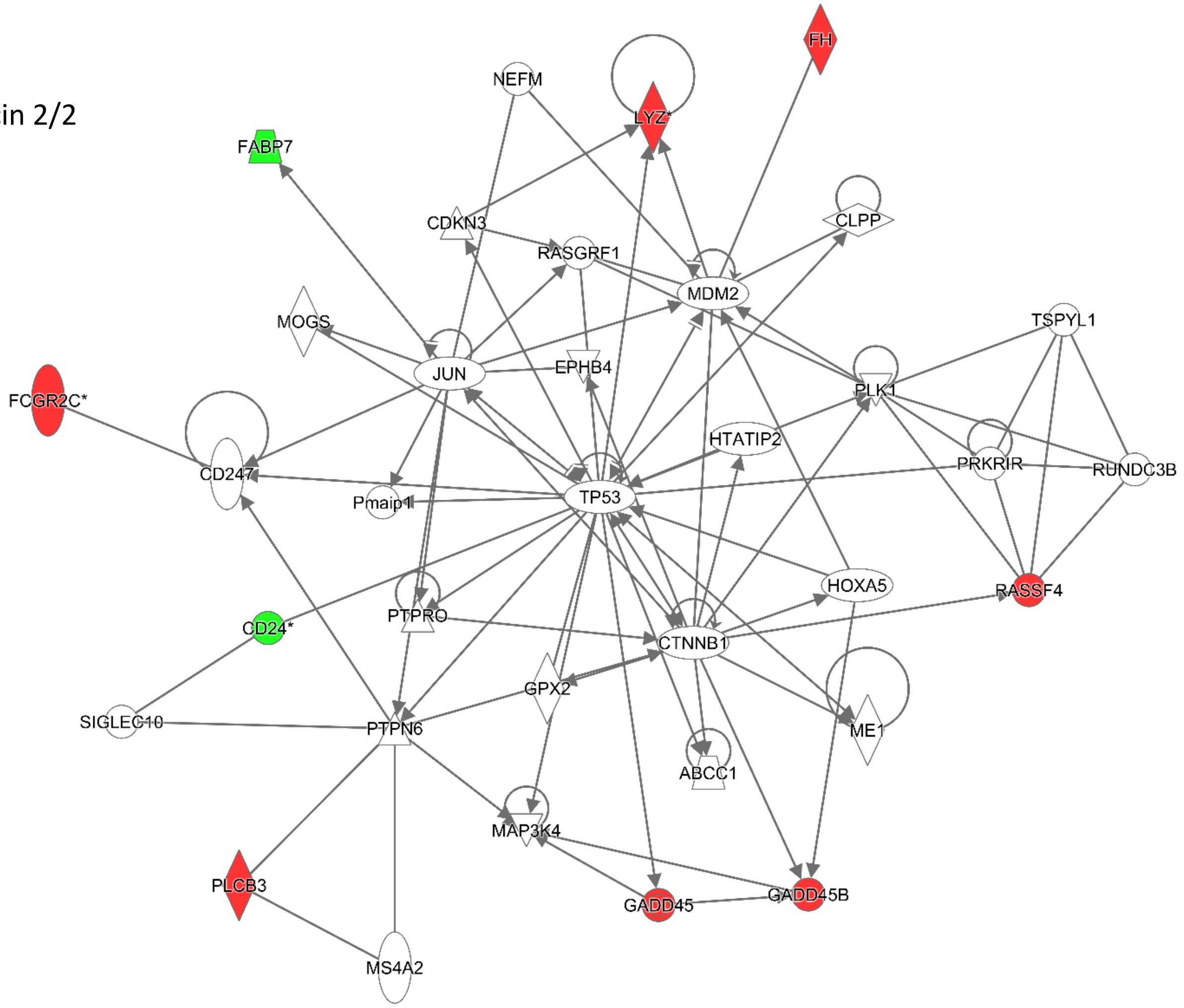

Supplementary Figure 4. IPA generated three gene networks (1/2 and 2/2) from the genes overlapping in the ionomycin and TBI signatures. Network 1 highlighted downregulation of tubulins and upregulation of NFE2L2. Color codes: red, upregulated genes; green, downregulated genes by ionomycin.

BRD-K75971499 1/1

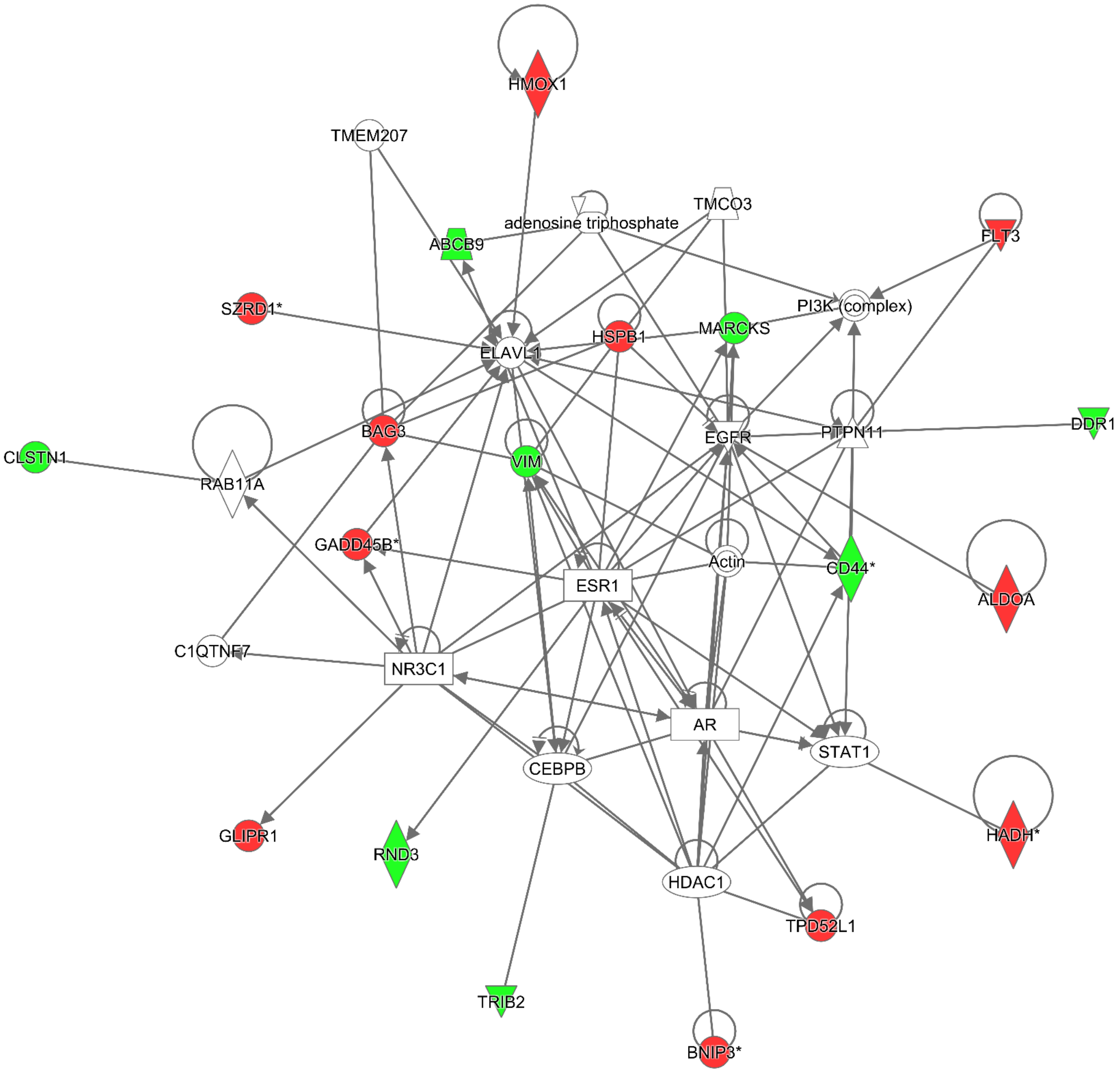

Supplementary Figure 5. IPA generated only one gene network (1/1) from genes overlapping in the BRD-K75971499 and TBI signatures. Network 1 highlighted CD44 downregulation. Color codes: red, upregulated genes; green, downregulated genes by BRD-K7597149.

BRD-K55260239 1/1

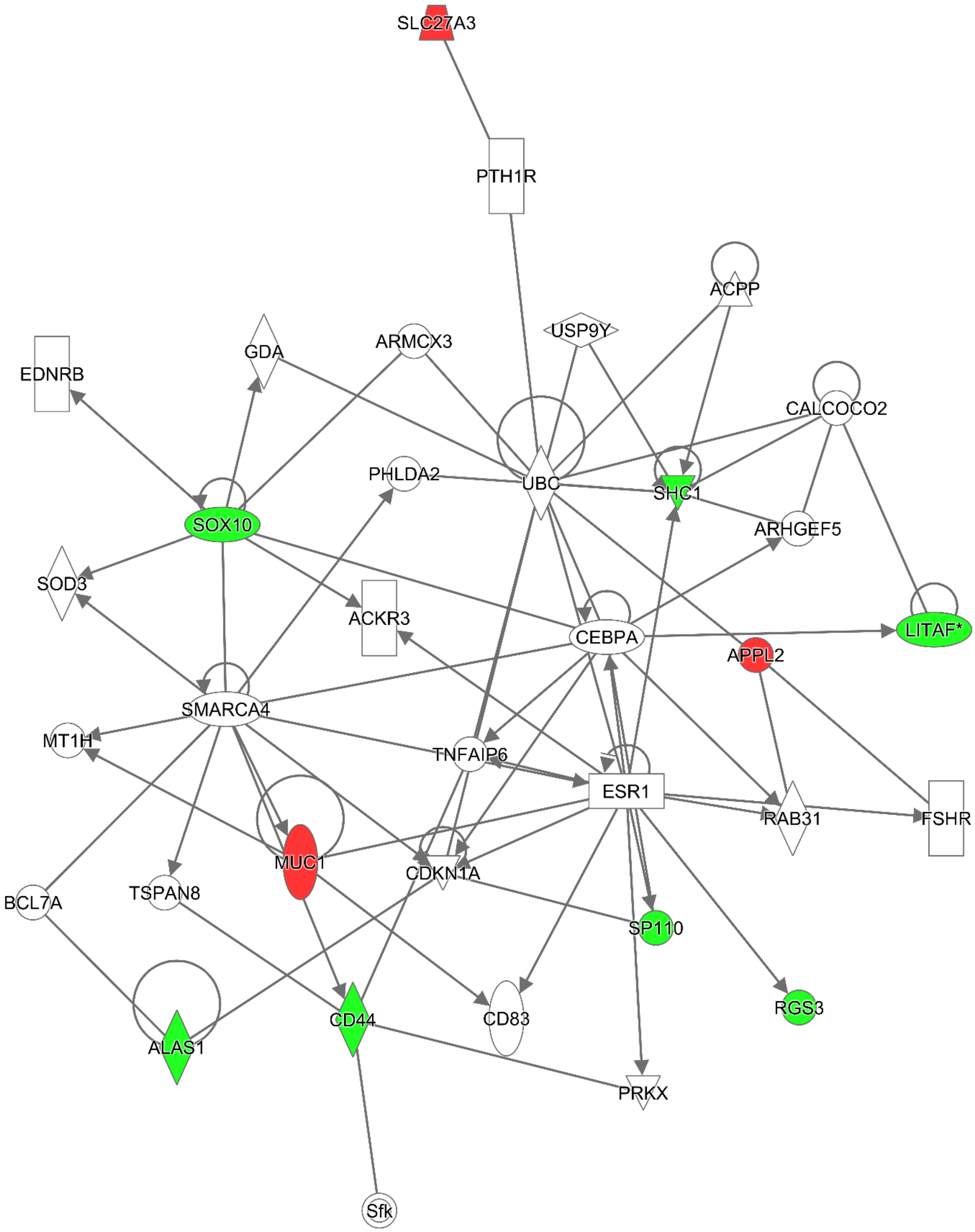

Supplementary Figure 6. IPA generated only one gene network (1/1) from genes overlapping in the BRD-K55260239 and TBI signatures. Network 1 highlighted CD44 downregulation. Color codes: red, upregulated genes; green, downregulated genes by BRD-K55260239.

SDZ-NKT-343 1/2

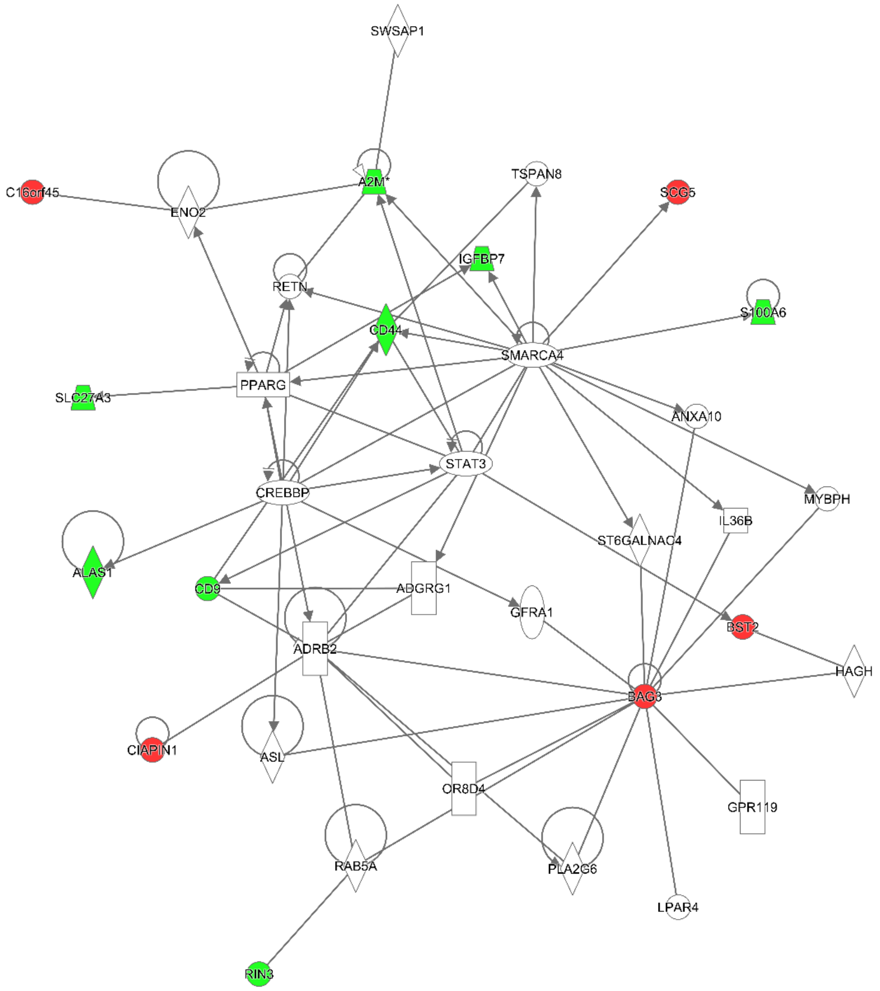

SDZ-NKT-343 2/2

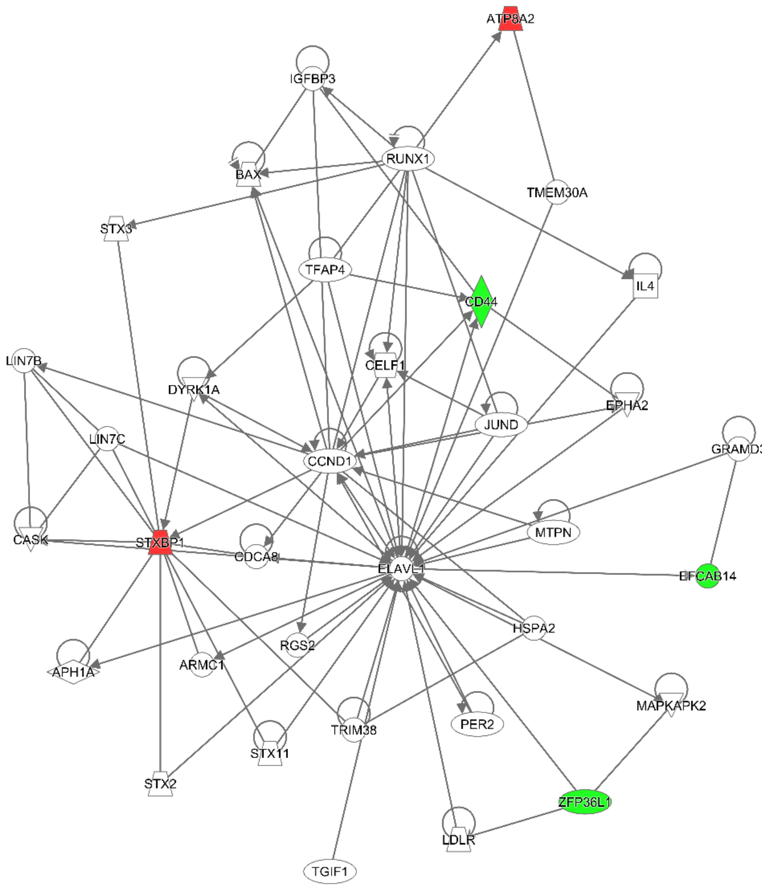

Supplementary Figure 7. IPA generated three gene networks (1/2 and 2/2) from genes overlapping in the SDZ-NKT-343 and TBI signatures. Network 1 highlighted CD44 downregulation. Color codes: red, upregulated genes; green, downregulated genes by SDZ-NKT-343.

STK-661558 1/2

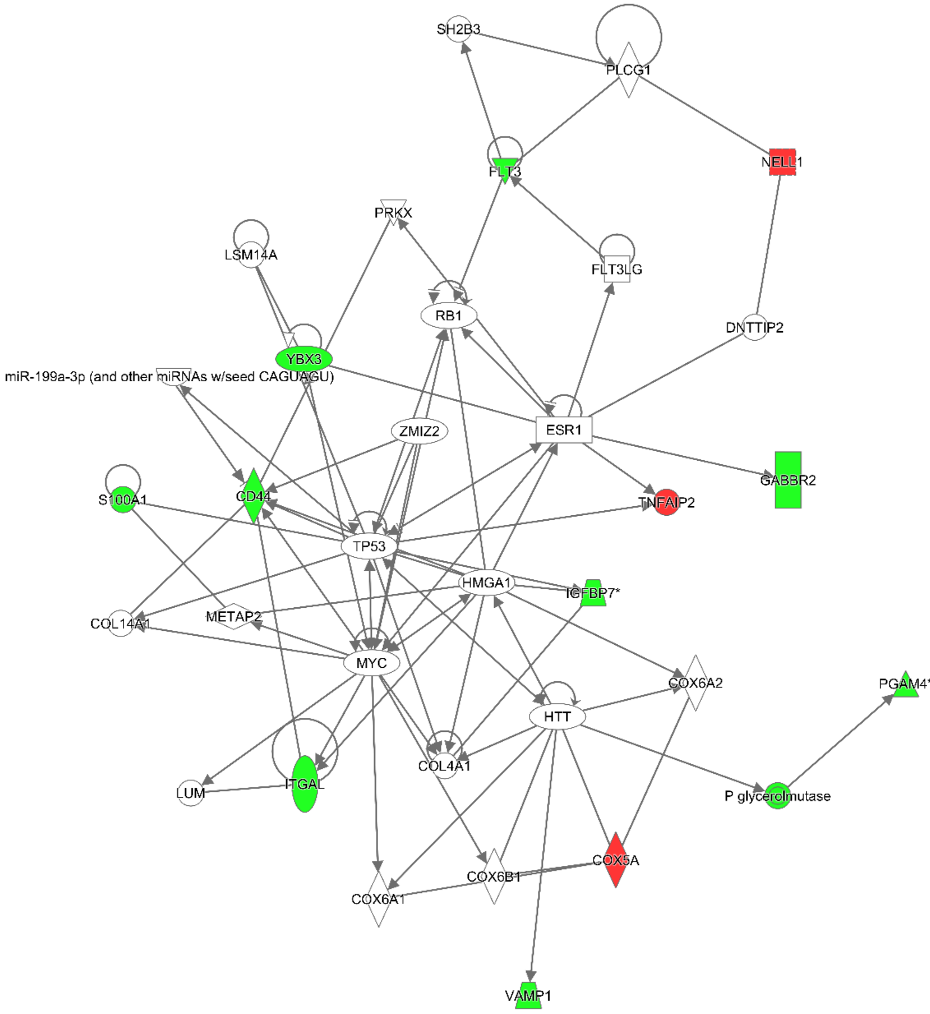

STK-661558 1/2

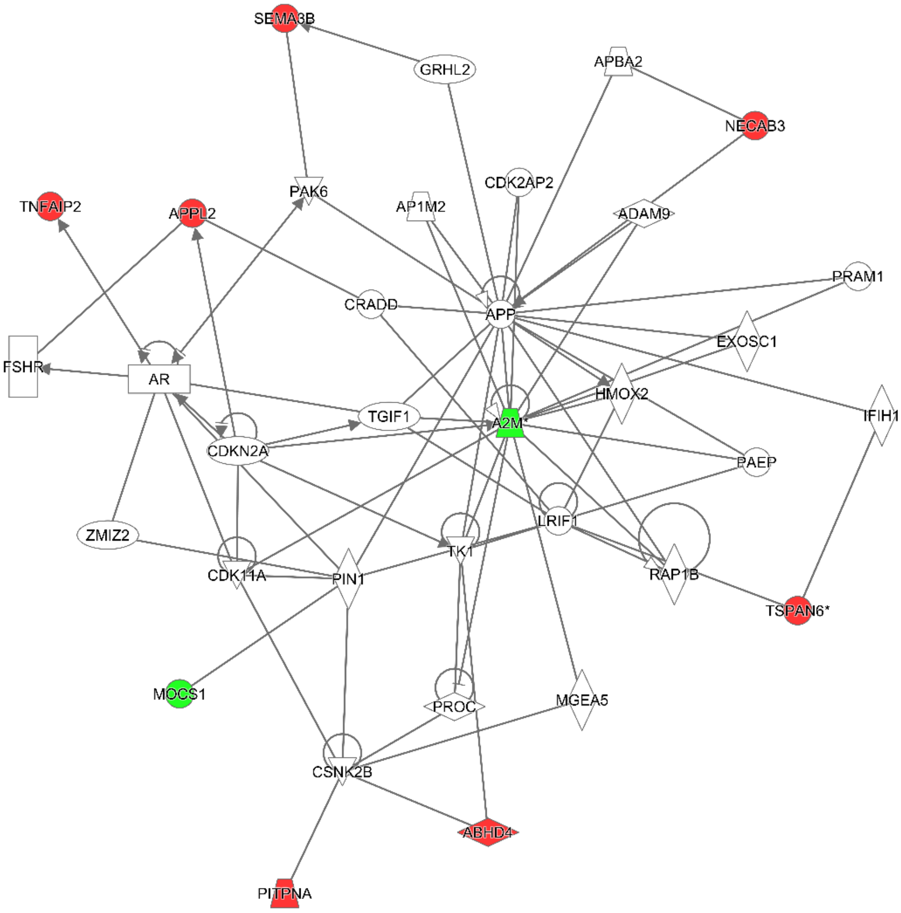

Supplementary Figure 8. IPA generated three gene networks (1/2 and 2/2) from genes overlapping in the STK-661558 and TBI signatures. Network 1 highlighted CD44 downregulation. Color codes: red, upregulated genes; green, downregulated genes by STK-661558.

sirolimus [illegible]

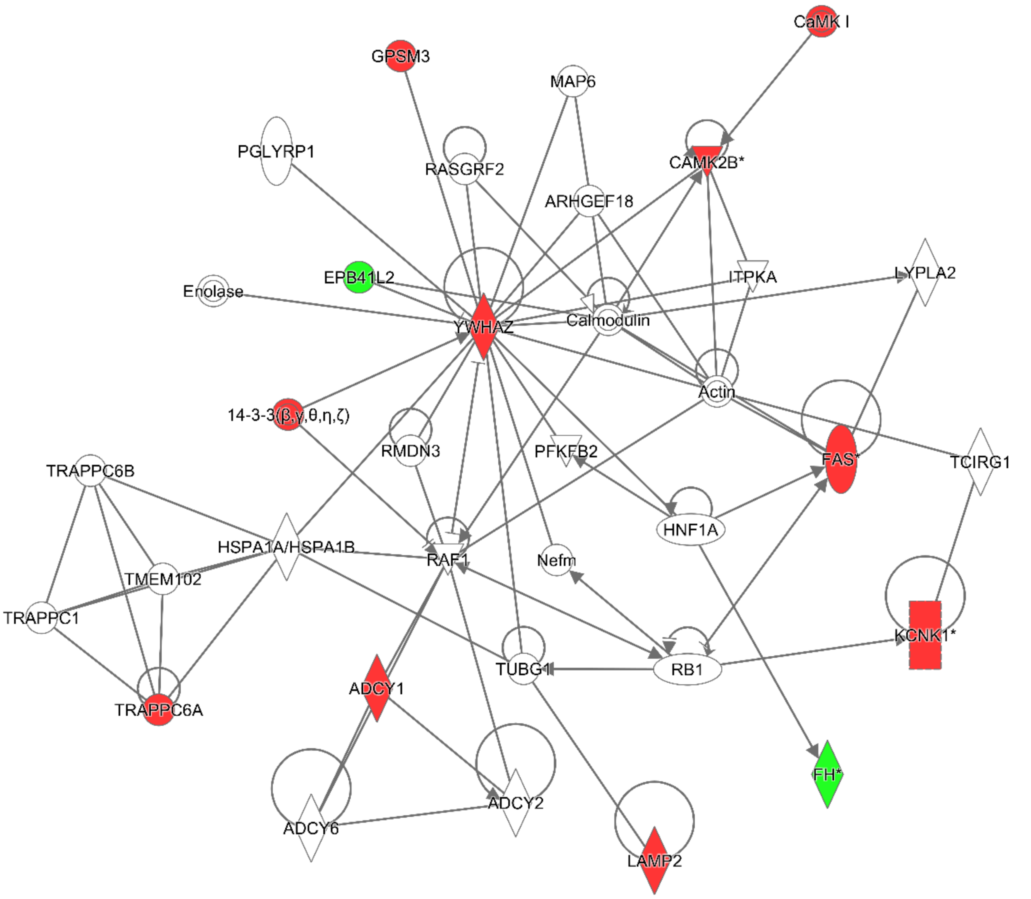

sirolimus 2/2

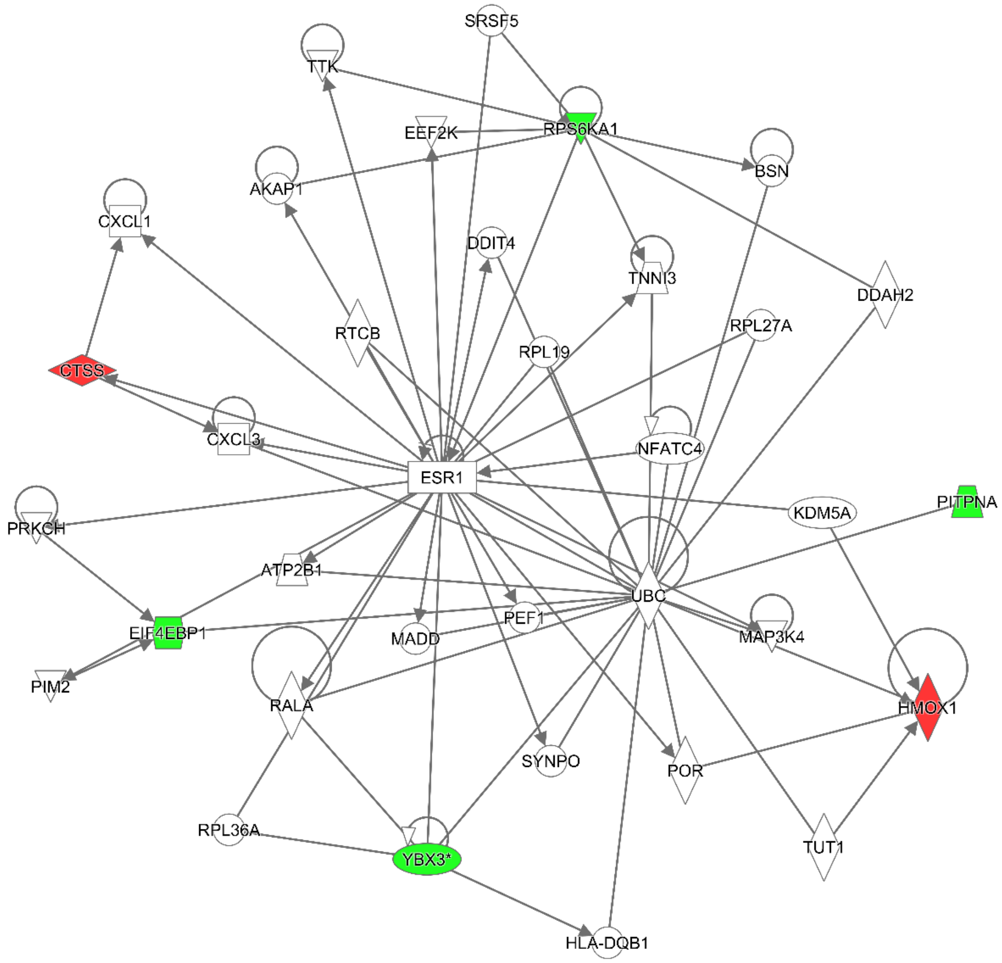

Supplementary Figure 9. IPA generated three gene networks (1/2 and 2/2) from genes overlapping in the sirolimus and TBI signatures. Network 1 highlighted YWHAZ upregulation. Color codes: red, upregulated genes; green, downregulated genes by sirolimus.

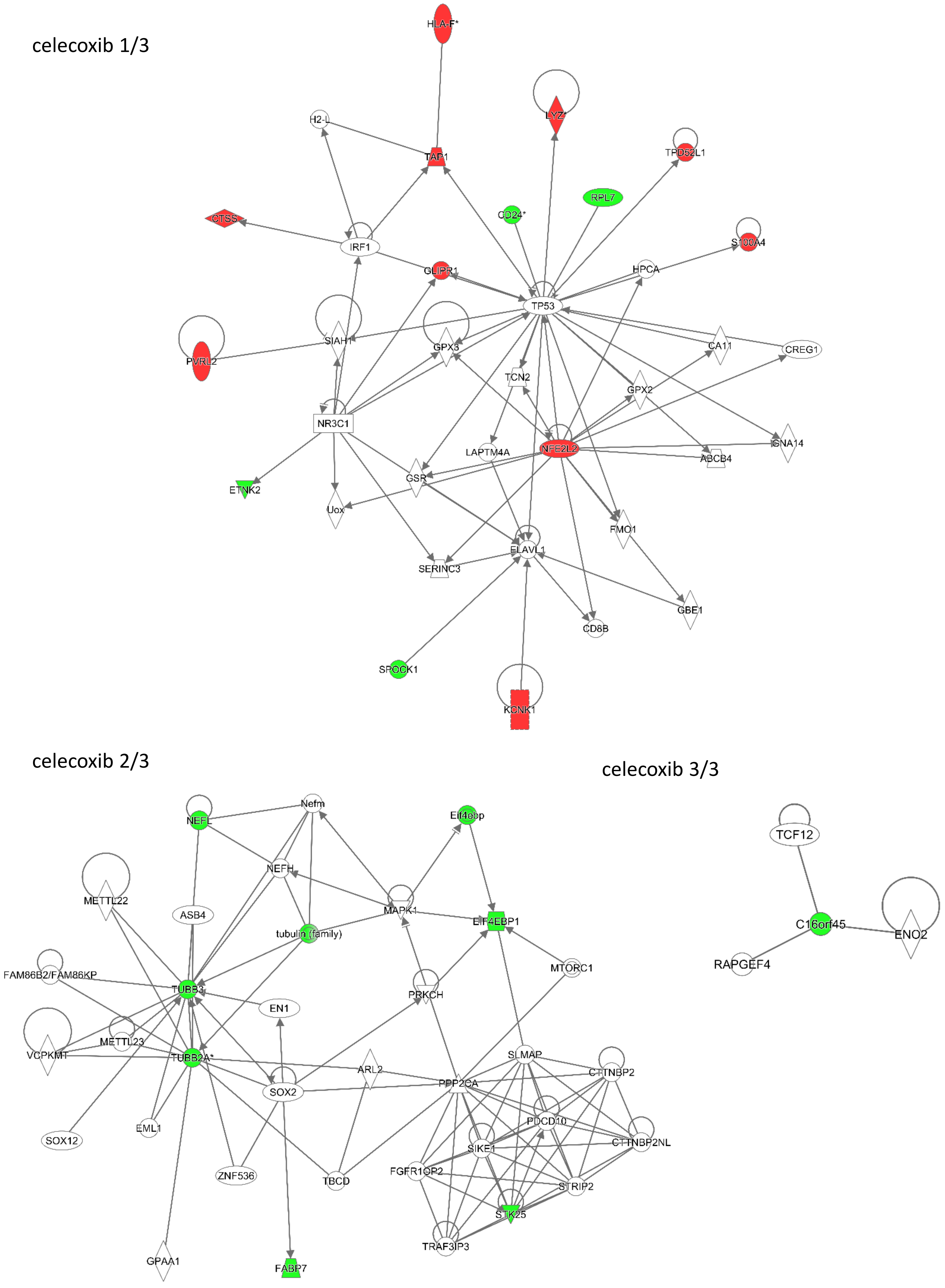

Supplementary Figure 10. IPA generated three gene networks (1/3, 2/3, and 3/3) from genes overlapping in the celecoxib and TBI signatures. Network 1 highlighted NFE2L2 and S100A4 upregulation. Network 2 highlighted tubulin downregulation. Color codes: red, upregulated genes; green, downregulated genes by celecoxib.

References

1. Pitkänen, A. & McIntosh, T. K. Animal models of post-traumatic epilepsy. *J. Neurotrauma* **23,** 241–61 (2006).

2. Echegoyen, J., Armstrong, C., Morgan, R. J. & Soltesz, I. Single application of a CB1 receptor antagonist rapidly following head injury prevents long-term hyperexcitability in a rat model. *Epilepsy Res.* **85,** 123–7 (2009).

3. Smith, D. C. *et al.* Recovery of function after vagus nerve stimulation initiated 24 hours after fluid percussion brain injury. *J. Neurotrauma* **23,** 1549–60 (2006).

4. Pitkänen, A. & Lukasiuk, K. Mechanisms of epileptogenesis and potential treatment targets. *Lancet. Neurol.* **10,** 173–86 (2011).
